# Changes in Anterior Chamber Parameters Measured by Pentacam in Coronavirus Disease 2019 Infection: A Longitudinal Study

**DOI:** 10.1155/2022/6828924

**Published:** 2022-02-01

**Authors:** Saeed Shokoohi-Rad, Mojtaba Abrishami, Elham Bakhtiari, Asma Payandeh, Hamid-Reza Heidarzadeh

**Affiliations:** Eye Research Center, Mashhad University of Medical Sciences, Mashhad, Iran

## Abstract

**Purpose:**

To evaluate changes in anterior segment parameters measured by imaging in coronavirus disease 2019 (COVID-19) infection.

**Methods:**

This longitudinal observational study included patients who recovered from COVID-19. All subjects exhibited a confirmed diagnosis of COVID-19 with a positive result of nasopharyngeal reverse transcription-polymerase chain reaction evaluation. Corneal Pentacam (Oculus, Dutenhofen, Germany) imaging was performed at least two weeks after recovery from systemic COVID-19. Measurements were repeated one and three months later.

**Results:**

A total of 20 patients, 11 (55%) of whom were females, with a mean age of 35.5 ± 7.1 years (age range: 25–51 years) recovered from nonhospitalized COVID-19 infection, were enrolled in this study. An increase in the keratometry mean front, keratometry mean back, cornea volume, and anterior chamber depth was observed in the longitudinal evaluation; however, they showed no statistical significance. The anterior chamber volume was statistically increased at three-month follow-up, compared to baseline (baseline: 177.8 ± 36.68 mm^3^; three months: 182.25 ± 39.58 mm^3^, *P*=0.048).

**Conclusion:**

COVID-19 infection may affect the cornea and anterior segment.

## 1. Introduction

Severe acute respiratory syndrome coronavirus 2 (SARS-CoV-2) caused an outbreak of unusual pneumonia with multiorgan damage worldwide [1]. Coronavirus disease 2019 (COVID-19) has various ophthalmic manifestations. Ocular surface, as well as corneal signs and symptoms, was reported, and the most prevalent manifestations were conjunctivitis and dry eye [2, 3]. Posterior segment involvements were numerous as cotton wool spots, retinal hemorrhages, dilated and tortuous vessels, decreased retinal microvasculature, ischemic retinal involvements, pachychoroid spectrum presentations, chorioretinitis, and optic nerve head involvement [4–7]. Furthermore, neuroinvasive actions of the virus were reported in previous studies [8, 9].

The presence of SARS-CoV-2 RNA and proteins was reported in the anterior chamber and corneal tissue following COVID-19 infection [10–13]. Moreover, some incompatible reports were on the presence of viruses on the ocular surface [14]. However, the long-term effects of COVID-19 infection on the anterior segment parameters and cornea are unclear. In posterior segment evaluations up to now, retinal thickness and vasculature have been affected in COVID-19 patients more than finding SARS-CoV-2 in retinal samples. A case-control study on 31 patients recovered from COVID-19 infection reported decreased vessel density in the superficial and deep retinal capillary plexus in the foveal and parafoveal regions [15]. In another study, remarkable changes of the macular region choroid were reported in a cross-sectional study on 34 patients recovered from COVID-19 infection [16]. Accordingly, this study aimed to evaluate the Pentacam parameters of the anterior segment and cornea to investigate the hypotheses of anterior segment changes secondary to uvea changes in COVID-19 infection.

## 2. Methods

### 2.1. Study Population

In this longitudinal study conducted in Khatam Eye Hospital, the participants were the hospital personnel who recovered from COVID-19 infection and came back to work. They were aged from 25 to 55 years, and the COVID-19 infection of all subjects was confirmed with nasopharyngeal swabs under reverse transcription-polymerase chain reaction test for SARS-CoV-2. The exclusion criteria were the presence of any ocular pathologic condition impairing visual function, any previous ocular surgery (except for refractive surgery more than six months ago), glaucoma or glaucoma suspect, diabetes mellitus, auto-immune diseases, pregnancy, breastfeeding, myopia more than −6.00 D or hyperopia more than +4.00 D, low-quality images, and best-corrected visual acuity less than 20/20. All subjects underwent a standard ophthalmic examination. At the time of becoming asymptomatic, at least two weeks after the diagnosis of the COVID-19 infection, the participants were asked for a baseline visit. Subsequently, examination and imaging were repeated after one and three months.

### 2.2. Imaging Procedures

Imaging was performed by a rotating Scheimpflug camera (Pentacam, OCULUS Optikgeräte, Germany). Keratometry mean front (*K*_*m*_ front), keratometry mean back (*K*_*m*_ back), cornea thinnest thickness (CCT), keratometry maximum front (*K*_max_ front), cornea volume (CV), anterior chamber volume (ACV), and anterior chamber depth (ACD) were extracted by the analyzer software.

### 2.3. Statistical Analysis

Statistical analysis was performed in SPSS software (version 16; SPSS Institute, Inc., Chicago, IL, USA) through ANOVA to compare the mean values between visits.

### 2.4. Ethical Considerations

Regarding the declaration of Helsinki, the imaging procedures were clarified for the participants, and written informed consent was obtained from each patient. The study protocol was approved by the Ethics Committee of the Human Research at Mashhad University of Medical Sciences, Mashhad, Iran (IR.MUMS.MEDICAL.REC.1399.597).

## 3. Results

A total of 20 healthy patients recovered from nonhospitalized COVID-19 infection were enrolled in this study. It is worth mentioning that the majority (*n* = 11; 55%) of the cases were female, and the mean age of the participants was 35.5 ± 7.12 years (age range: 25–51 years). The mean values of the Km front were 42.4 ± 2 D, 42.45 ± 1.94 D, and 42.6 ± 2.07 D at baseline, as well as one and three months later, respectively. This increase was not statically significant (*P*=0.19), and there was no significant difference between the two visits (Table 1). The mean CTT values were 524.2 ± 40.05, 523.8 ± 39.74, and 526 ± 39.58 *μ*m at baseline, as well as one and three months later, respectively. It should be noted that there were no significant changes during the time (*P*=0.257) and between the visits (Table 1).

Furthermore, the mean CV values were 59.68 ± 4.22, 59.78 ± 4.24, and 59.83 ± 4.21 at baseline, as well as one and three months later, respectively. This increase was not significant (*P*=0.542), and there were no significant changes between the two visits (Table 1). The mean ACV values were 177.8 ± 36.68, 180.8 ± 44.91, and 182.25 ± 39.58 mm^3^ at baseline, as well as one and three months later, with no significant increase (*P*=0.13). However, there was a significant increase three months later, compared to the baseline (*P*=0.048) (Table 1). Other parameter changes are depicted in Table 1.

## 4. Discussion

This study evaluated seven anterior chamber and cornea Pentacam parameters. The mean *K*_*m*_ front, *K*_*m*_ back, CV, ACD, and ACV values showed an increase during three months; however, these increases were not statically significant. The mean CTT and *K*_max_ front showed no significant changes in the period of the study. The comparison of these parameter values between different visits showed no significance, except for ACV. SARS-CoV-2 virus presence has been reported in the anterior chamber and cornea tissue [10–13]. Yan et al. reported the presence of nucleocapsid protein antigens on the cells of the conjunctiva, iris, and trabecular meshwork of a patient with a COVID-19 infection, and these antigens were absent on the specimens from the control patient [10]. Casagrande et al. in a case series of 11 patients with COVID-19 viremia reported the presence of viral genomic (55%) and subgenomic (67%) RNA of SARS-CoV-2 in the cornea [11]. Similarly, Sawant et al. reported that the positivity rate of SARS-CoV-2 RNA was about 13% in 132 ocular tissues from 33 surgical intended donors [12]. Koo et al. in a prospective cross-sectional study on 31 aqueous humor samples found that the SARS-CoV-2 RNA was in 19.4% of the samples [13]. However, Bayyoud et al. reported that no SARS-CoV-2 RNA was detected in the conjunctiva, anterior chamber fluid, and corneal tissues of 10 eyes of five COVID-19 patients [17]. SARS-CoV-2 uses the angiotensin-converting enzyme 2 (ACE2) for entry into target cells, and endothelial cells express abundant ACE2; moreover, the expression of ACE2 has been detected in the human aqueous humor [18, 19]. The direct and indirect effects of SARS-CoV-2 on the anterior chamber and cornea in the long term are unknown. Due to the possibility of SARS-CoV-2 presence in the anterior chamber and cornea, as well as the expression of ACE2 in the aqueous humor, it was decided to evaluate the anterior chamber and cornea Pentacam parameter changes in participants recovered from COVID-19 infection in a longitudinal study.

In total, 40 eyes of 20 patients with no ocular pathology were investigated after recovery from COVID-19 infection with Pentacam imaging. All patients were asymptomatic and had no ocular signs or symptoms. The mean *K*_*m*_ front, *K*_*m*_ back, CV, ACD, and ACV showed an increase; however, they were not statically significant. On the other hand, these increases may get significant in a more extended time and need more extended studies on a larger number of participants. In the same line, there were no significant changes in the mean CTT and *K*_max_. The only statistically significant change in this study was the increased value of ACV after three months, compared to baseline (*P*=0.048). An increase was noticed in the ACD, which could be significant in the long term, and it could be secondary to anterior segment vascular changes.

In conclusion, the effect of COVID-19 infection on the anterior chamber and cornea was investigated in this study. The increase in some anterior chamber and cornea Pentacam parameters was not significant, except for the ACV; however, the reversibility of this effect and evaluation of other parameters need further studies on larger populations and follow-up of these patients. Our participants were from a small group of otherwise healthy nonhospitalized patients with no complications following COVID-19 infection. This point was one of our study limitations. The subjects were followed up just for three months, and more follow-ups could specify whether these changes are temporary/permanent and progressive. It was the first time that anterior segment changes in COVID-19 patients were evaluated with anterior segment modalities. Further studies to obtain accumulative evidence on COVID-19 effects on anterior segment parameters may lead to clinical application in elective intraocular surgeries, such as considering parameters in intraocular lens (IOL) calculation for phakic IOL or phacoemulsification candidates with a history of COVID-19 infection.

Further studies are recommended to evaluate iris vascularity in COVID-19 patients with anterior segment optical coherence tomography angiography and use more accurate imaging modalities than Pentacam, such as anterior segment optical coherence tomography, to evaluate anterior segment parameters.

## Figures and Tables

**Table 1:** Pentacam parameter mean values in different visits and comparison between different visits. SD: standard deviation; *K*_*m*_ front: keratometry mean front; *K*_*m*_ back: keratometry mean back; CTT: cornea thinnest thickness; *K*_max_ front: keratometry maximum front; CV: cornea volume; ACV: anterior chamber volume; ACD: anterior chamber depth.

Parameters	Baseline, mean ± SD	1 month, mean ± SD	3 months, mean ± SD	*P* value	Baseline vs. 1-month mean difference ± SD (*P* value)	1-month vs. 3-month mean difference ± SD (*P* value)	Baseline vs. 3-month mean difference ± SD (*P* value)
*K*_*m*_ front	42.4 ± 2	42.45 ± 1.94	42.6 ± 2.07	0.19	−0.05 ± 0.03 (0.46)	−0.15 ± 0.13 (0.86)	−0.2 ± 0.13 (0.44)
*K*_*m*_ back	−6.21 ± 0.26	−6.22 ± 0.27	−6.23 ± 0.24	0.47	0 ± 0.01 (1)	0.01 ± 0.01 (1)	0.01 ± 0.01 (0.55)
CTT	524.2 ± 40.5	523.8 ± 39.74	526 ± 39.58	0.25	0.4 ± 1.41 (1)	−2.2 ± 1.41 (0.40)	−1.8 ± 1.36 (0.60)
*K*_max_ front	43.91 ± 1.61	43.88 ± 1.59	43.94 ± 1.51	0.55	0.03 ± 0.05 (1)	−0.06 ± 0.05 (0.77)	−0.03 ± 0.06 (1)
CV	59.68 ± 4.22	59.78 ± 4.24	59.83 ± 4.21	0.54	−0.1 ± 0.16 (1)	−0.04 ± 0.1 (1)	−0.15 ± 0.13 (0.88)
ACV	177.8 ± 36.68	180.8 ± 44.91	182.25 ± 39.58	0.13	−3 ± 2.68 (0.83)	−1.45 ± 2.08 (1)	−4.45 ± 1.68 (0.04)
ACD	2.99 ± 0.37	2.99 ± 0.39	3 ± 0.38	0.57	0 ± 0.01 (1)	0 ± 0.01 (1)	−0.01 ± 0.01 (0.84)

## Data Availability

The datasets used during the current study are available from the corresponding author on reasonable request.
